# (2*Z*,*N*′*E*)-*N*′-[(2-Hy­droxy-1-naphth­yl)methyl­idene]furan-2-carbohydrazonic acid

**DOI:** 10.1107/S1600536810027935

**Published:** 2010-07-21

**Authors:** Rahman Bikas, Hassan Hosseini Monfared, Keyvan Bijanzad, Ahmet Koroglu, Canan Kazak

**Affiliations:** a Department of Chemistry, Zanjan University, 45195-313 Zanjan, Iran; b Faculty of Chemistry, Iran University of Science and Technology (IUST), 16846 Tehran, Iran; c Department of Physics, Faculty of Arts and Sciences, Ondokuz Mayis University, 55019 Kurupelit, Samsun, Turkey

## Abstract

In the title compound, C_16_H_12_N_2_O_3_, the dihedral angle between the mean planes of the naphthalene ring system and the furan ring is 21.3 (6)°. The mol­ecular structure is stabilized by an intra­molecular O—H⋯N hydrogen bond, which generates an *S*(6) graph-set motif.

## Related literature

For historical background to aroylhydrazones, see: Arapov *et al.* (1987 ▶); Pickart *et al.* (1983 ▶); Offe *et al.* (1952 ▶); Nagaraju *et al.* (2009 ▶); Ghosh *et al.* (2007 ▶). For related structures, see: Monfared *et al.* (2010 ▶); Ali *et al.* (2005 ▶); Qian *et al.* (2006 ▶); Tarafder *et al.* (2002 ▶); Prathapachandra Kurup & Bessy Raj (2007 ▶). For graph-set analysis of hydrogen-bond networks, see: Bernstein *et al.* (1995 ▶); Etter *et al.* (1990 ▶). For bond-length data, see: Allen *et al.* (1987 ▶).
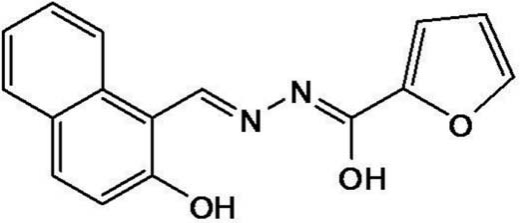


## Experimental

### 

#### Crystal data

C_16_H_12_N_2_O_3_
*M* *_r_* = 280.28
Orthorhombic, 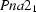

*a* = 9.7427 (8) Å
*b* = 21.4182 (8) Å
*c* = 6.445 (2) Å
*V* = 1344.8 (4) Å^3^
*Z* = 4
Mo *K*α radiation
μ = 0.10 mm^−1^
*T* = 293 K
0.31 × 0.27 × 0.15 mm

#### Data collection

STOE IPDS 2 diffractometer
Absorption correction: integration (*X-RED32*; Stoe & Cie, 2002 ▶) *T*_min_ = 0.970, *T*_max_ = 0.985
7278 measured reflections
1440 independent reflections
944 reflections with *I* > 2σ(*I*)
*R*_int_ = 0.097

#### Refinement

*R*[*F* ^2^ > 2σ(*F* ^2^)] = 0.052
*wR*(*F* ^2^) = 0.095
*S* = 1.02
1440 reflections
191 parameters
1 restraint
H-atom parameters constrained
Δρ_max_ = 0.18 e Å^−3^
Δρ_min_ = −0.13 e Å^−3^

### 

Data collection: *X-AREA* (Stoe & Cie, 2002 ▶); cell refinement: *X-AREA*; data reduction: *X-RED32* (Stoe & Cie, 2002 ▶); program(s) used to solve structure: *SHELXS97* (Sheldrick, 2008 ▶); program(s) used to refine structure: *SHELXL97* (Sheldrick, 2008 ▶); molecular graphics: *ORTEP-3 for Windows* (Farrugia, 1997 ▶); software used to prepare material for publication: *WinGX* (Farrugia, 1999 ▶).

## Supplementary Material

Crystal structure: contains datablocks global, I. DOI: 10.1107/S1600536810027935/jj2034sup1.cif

Structure factors: contains datablocks I. DOI: 10.1107/S1600536810027935/jj2034Isup2.hkl

Additional supplementary materials:  crystallographic information; 3D view; checkCIF report

## Figures and Tables

**Table 1:** Hydrogen-bond geometry (Å, °)

*D*—H⋯*A*	*D*—H	H⋯*A*	*D*⋯*A*	*D*—H⋯*A*
O1—H1⋯N1	0.82	1.84	2.565 (5)	146

**Fig. 1.**
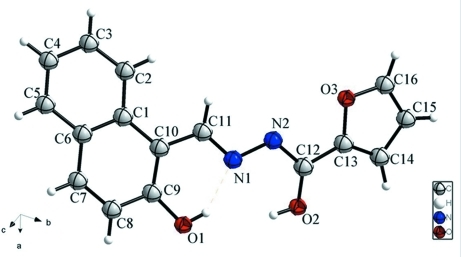
The molecular structure of the title compound, C16H12N2O3, with atom labels and anisotropic displacement ellipsoids (drawn at 30% probability level) for non-H atoms.

**Fig. 2.**
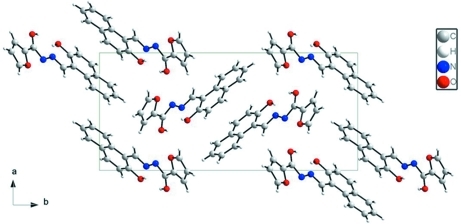
View of the unit cell of the title compound, C16H12N2O3, viewed along [110].

## supplementary crystallographic information

### Comment

Hydrazone ligands derived from the condensation of aliphatic acid hydrazides or aromatic acid hydrazides with aromatic 2-hydroxy carbonyl compounds are important tridentate O, N,O-donor ligands. These compounds, due to their facile keto-enol tautomerization and the availability of several potential donor sites, can coordinate to metals.  Furthermore, the possibility of tautomerism makes their study interesting (Ghosh *et al.*, 2007). Hydrazones have wide spread applications in coordination, analytical and bioinorganic chemistry, and display magnetic, electronic, NLO and fluorescent properties in biologically active compounds (Prathapachandra Kurup *et al.*, 2007). They find applications in the treatment of diseases such as anti-tumor, tuberculosis, leprosy and mental disorder (Nagaraju *et al.*, 2009). As part of our studies on the synthesis and characterization of aroylhydrazone derivatives, we report here the crystal structure of C_16_H_12_N_2_O_3_.

In the title compound, C_16_H_12_N_2_O_3_, the dihedral angle between the mean planes of the naphthalene and furan rings is 21.3 (6)° (Fig.1).  The angle formed between the menn planes of the naphthalene substituted hydroxy group (C11/C10/C1/O1/H1) and the 2-carbohydrazonic acid furan substituted hydroxy group (N1/N2/C12/O2/H22) is 17.9 (1)°. Bond distances and angles are in normal ranges (Allen *et al.*, 1987). Crystal packing is stabilized by O1—H1···N1 intramolecular hydrogen bonds which form an S_1_^1^(6) graph-set motif (Bernstein *et al.*, 1995), Etter *et al.*, 1990), (Fig. 2).

### Experimental

All reagents were commercially available and used as received. A methanol (10 ml) solution of 2-hydroxy-1-naphtaldehyde (1.5 mmol) was drop-wise added to a methanol solution (10 ml) of 2-furanecarboxylic acid hydrazide (1.5 mmol), and the mixture was refluxed for 3 h. Then the solution was evaporated on a steam bath to 5 cm^3^ and cooled to room temperature. Yellow precipitates of the title compound were separated and filtered off, washed with 3 ml of cooled methanol and then dried in air. X-ray quality crystals of the title compound were obtained from methanol by slow solvent evaporation. Yield: 82%, mp 197-198 °C.

### Refinement

The hydroxyl hydrogen atoms were located by Fourier analysis and refined using the riding model with d(O—H) = 0.82Å [*U*_iso_(H) = 1.5*U*_eq_(O)].  C-bonded H atoms were positioned geometrically (C—H = 0.93 Å) and treated as riding on their parent atoms [*U*_iso_(H) = 1.2*U*_eq_(C)].

### Crystal data

**Table:**

C_16_H_12_N_2_O_3_	*D*_x_ = 1.384 Mg m^−^^3^
*M**_r_* = 280.28	Melting point = 470–471 K
Orthorhombic, *P**n**a*2_1_	Mo *K*α radiation, λ = 0.71073 Å
Hall symbol: P 2c -2n	Cell parameters from 8863 reflections
*a* = 9.7427 (8) Å	θ = 1.9–27.1°
*b* = 21.4182 (8) Å	µ = 0.10 mm^−^^1^
*c* = 6.445 (2) Å	*T* = 293 K
*V* = 1344.8 (4) Å^3^	Prism, colourless
*Z* = 4	0.31 × 0.27 × 0.15 mm
*F*(000) = 584	

### Data collection

**Table:**

STOE IPDS 2 diffractometer	1440 independent reflections
Radiation source: fine-focus sealed tube	944 reflections with *I* > 2σ(*I*)
plane graphite	*R*_int_ = 0.097
Detector resolution: 6.67 pixels mm^-1^	θ_max_ = 26.0°, θ_min_ = 1.9°
rotation method scans	*h* = −12→10
Absorption correction: integration (*X-RED32*; Stoe & Cie, 2002)	*k* = −26→24
*T*_min_ = 0.970, *T*_max_ = 0.985	*l* = −7→7
7278 measured reflections	

### Refinement

**Table:**

Refinement on *F*^2^	Secondary atom site location: difference Fourier map
Least-squares matrix: full	Hydrogen site location: inferred from neighbouring sites
*R*[*F*^2^ > 2σ(*F*^2^)] = 0.052	H-atom parameters constrained
*wR*(*F*^2^) = 0.095	*w* = 1/[σ^2^(*F*_o_^2^) + (0.031*P*)^2^] where *P* = (*F*_o_^2^ + 2*F*_c_^2^)/3
*S* = 1.02	(Δ/σ)_max_ < 0.001
1440 reflections	Δρ_max_ = 0.18 e Å^−^^3^
191 parameters	Δρ_min_ = −0.13 e Å^−^^3^
1 restraint	Extinction correction: *SHELXL97* (Sheldrick, 2008), Fc^*^=kFc[1+0.001xFc^2^λ^3^/sin(2θ)]^-1/4^
Primary atom site location: structure-invariant direct methods	Extinction coefficient: 0.0063 (17)

### Special details

**Table:**

Experimental. 12912 Friedel pairs have been merged
Geometry. All e.s.d.'s (except the e.s.d. in the dihedral angle between two l.s. planes) are estimated using the full covariance matrix. The cell e.s.d.'s are taken into account individually in the estimation of e.s.d.'s in distances, angles and torsion angles; correlations between e.s.d.'s in cell parameters are only used when they are defined by crystal symmetry. An approximate (isotropic) treatment of cell e.s.d.'s is used for estimating e.s.d.'s involving l.s. planes.
Refinement. Refinement of *F*^2^ against ALL reflections. The weighted *R*-factor *wR* and goodness of fit *S* are based on *F*^2^, conventional *R*-factors *R* are based on *F*, with *F* set to zero for negative *F*^2^. The threshold expression of *F*^2^ > σ(*F*^2^) is used only for calculating *R*-factors(gt) *etc*. and is not relevant to the choice of reflections for refinement. *R*-factors based on *F*^2^ are statistically about twice as large as those based on *F*, and *R*- factors based on ALL data will be even larger.

### Fractional atomic coordinates and isotropic or equivalent isotropic displacement parameters (Å^2^)

**Table:**

	*x*	*y*	*z*	*U*_iso_*/*U*_eq_	
C1	0.2976 (5)	0.54675 (18)	0.6316 (7)	0.0451 (11)	
C2	0.2057 (5)	0.5256 (2)	0.4789 (8)	0.0522 (12)	
H2	0.1969	0.5479	0.3559	0.063*	
C3	0.1290 (6)	0.4730 (2)	0.5070 (8)	0.0706 (16)	
H3	0.0688	0.4601	0.4036	0.085*	
C4	0.1400 (7)	0.4384 (2)	0.6892 (10)	0.0786 (18)	
H4	0.0883	0.4022	0.7065	0.094*	
C5	0.2262 (6)	0.4574 (2)	0.8410 (10)	0.0714 (16)	
H5	0.2320	0.4347	0.9636	0.086*	
C6	0.3079 (5)	0.51178 (19)	0.8155 (7)	0.0499 (12)	
C7	0.4022 (6)	0.5297 (2)	0.9729 (7)	0.0591 (14)	
H7	0.4051	0.5078	1.0974	0.071*	
C8	0.4882 (5)	0.5788 (2)	0.9420 (8)	0.0552 (13)	
H8	0.5516	0.5897	1.0436	0.066*	
C9	0.4814 (5)	0.61298 (19)	0.7575 (7)	0.0474 (11)	
C10	0.3861 (5)	0.6002 (2)	0.6042 (6)	0.0406 (10)	
C11	0.3758 (5)	0.63934 (18)	0.4226 (6)	0.0440 (11)	
H11	0.3027	0.6340	0.3314	0.053*	
C12	0.5461 (5)	0.7550 (2)	0.1419 (7)	0.0488 (11)	
C13	0.5167 (5)	0.78815 (19)	−0.0481 (7)	0.0513 (12)	
C14	0.5895 (6)	0.8270 (2)	−0.1646 (8)	0.0631 (14)	
H14	0.6764	0.8425	−0.1343	0.076*	
C15	0.5117 (7)	0.8404 (3)	−0.3412 (9)	0.0810 (19)	
H15	0.5371	0.8661	−0.4511	0.097*	
C16	0.3952 (7)	0.8095 (3)	−0.3215 (9)	0.0783 (18)	
H16	0.3240	0.8102	−0.4178	0.094*	
N1	0.4666 (4)	0.68174 (16)	0.3852 (6)	0.0488 (10)	
N2	0.4425 (4)	0.71739 (15)	0.2109 (6)	0.0495 (10)	
O1	0.5751 (3)	0.65972 (14)	0.7404 (5)	0.0632 (10)	
H1	0.5641	0.6779	0.6296	0.095*	
O2	0.6585 (3)	0.76014 (14)	0.2278 (5)	0.0658 (10)	
H22	0.6598	0.7385	0.3328	0.099*	
O3	0.3934 (3)	0.77680 (15)	−0.1422 (6)	0.0675 (10)	

### Atomic displacement parameters (Å^2^)

**Table:**

	*U*^11^	*U*^22^	*U*^33^	*U*^12^	*U*^13^	*U*^23^
C1	0.050 (3)	0.037 (2)	0.048 (3)	0.008 (2)	0.011 (2)	0.001 (2)
C2	0.052 (3)	0.048 (3)	0.056 (3)	0.002 (2)	0.008 (3)	−0.001 (2)
C3	0.075 (4)	0.056 (3)	0.081 (4)	−0.017 (3)	0.005 (3)	−0.006 (3)
C4	0.084 (5)	0.052 (3)	0.100 (5)	−0.015 (3)	0.023 (4)	0.005 (4)
C5	0.076 (4)	0.058 (3)	0.080 (4)	0.001 (3)	0.015 (4)	0.009 (3)
C6	0.052 (3)	0.040 (2)	0.057 (3)	0.010 (2)	0.011 (2)	0.002 (2)
C7	0.073 (4)	0.057 (3)	0.047 (3)	0.023 (3)	0.002 (3)	0.013 (2)
C8	0.055 (3)	0.060 (3)	0.050 (3)	0.016 (3)	−0.007 (2)	−0.011 (3)
C9	0.046 (3)	0.042 (2)	0.054 (3)	0.008 (2)	−0.001 (2)	−0.003 (2)
C10	0.036 (3)	0.043 (2)	0.043 (2)	0.011 (2)	0.003 (2)	0.001 (2)
C11	0.037 (3)	0.047 (2)	0.049 (3)	0.001 (2)	0.000 (2)	−0.002 (2)
C12	0.036 (3)	0.054 (3)	0.056 (3)	−0.002 (2)	0.005 (2)	−0.008 (2)
C13	0.044 (3)	0.047 (2)	0.063 (3)	0.000 (2)	0.007 (3)	0.005 (2)
C14	0.059 (3)	0.060 (3)	0.070 (3)	−0.018 (3)	0.012 (3)	0.009 (3)
C15	0.087 (5)	0.075 (4)	0.081 (4)	0.011 (4)	0.027 (4)	0.034 (3)
C16	0.060 (4)	0.087 (4)	0.088 (5)	0.022 (3)	0.011 (3)	0.033 (4)
N1	0.038 (2)	0.050 (2)	0.058 (3)	−0.002 (2)	0.0096 (18)	0.0013 (19)
N2	0.043 (2)	0.0485 (19)	0.057 (2)	−0.0082 (18)	0.0117 (19)	0.0134 (19)
O1	0.056 (2)	0.060 (2)	0.074 (2)	−0.0063 (18)	−0.0109 (19)	−0.0017 (18)
O2	0.056 (2)	0.078 (2)	0.064 (2)	−0.0186 (18)	0.001 (2)	0.0063 (19)
O3	0.040 (2)	0.078 (2)	0.085 (3)	0.0037 (18)	0.0047 (19)	0.0323 (19)

### Geometric parameters (Å, °)

**Table:**

C1—C2	1.406 (6)	C10—C11	1.443 (6)
C1—C6	1.406 (6)	C11—N1	1.291 (5)
C1—C10	1.444 (6)	C11—H11	0.9300
C2—C3	1.364 (7)	C12—O2	1.232 (5)
C2—H2	0.9300	C12—N2	1.366 (5)
C3—C4	1.393 (8)	C12—C13	1.444 (6)
C3—H3	0.9300	C13—C14	1.326 (6)
C4—C5	1.352 (8)	C13—O3	1.367 (5)
C4—H4	0.9300	C14—C15	1.398 (7)
C5—C6	1.420 (7)	C14—H14	0.9300
C5—H5	0.9300	C15—C16	1.321 (8)
C6—C7	1.422 (7)	C15—H15	0.9300
C7—C8	1.359 (7)	C16—O3	1.352 (6)
C7—H7	0.9300	C16—H16	0.9300
C8—C9	1.397 (6)	N1—N2	1.379 (5)
C8—H8	0.9300	O1—H1	0.8200
C9—O1	1.359 (5)	O2—H22	0.8200
C9—C10	1.384 (6)		
			
C2—C1—C6	117.6 (4)	C9—C10—C11	120.7 (4)
C2—C1—C10	123.4 (4)	C9—C10—C1	118.1 (4)
C6—C1—C10	118.9 (4)	C11—C10—C1	121.2 (4)
C3—C2—C1	121.5 (5)	N1—C11—C10	120.8 (4)
C3—C2—H2	119.3	N1—C11—H11	119.6
C1—C2—H2	119.3	C10—C11—H11	119.6
C2—C3—C4	120.6 (5)	O2—C12—N2	124.3 (4)
C2—C3—H3	119.7	O2—C12—C13	120.9 (4)
C4—C3—H3	119.7	N2—C12—C13	114.8 (4)
C5—C4—C3	119.8 (5)	C14—C13—O3	109.3 (4)
C5—C4—H4	120.1	C14—C13—C12	133.0 (5)
C3—C4—H4	120.1	O3—C13—C12	117.6 (4)
C4—C5—C6	120.8 (6)	C13—C14—C15	107.5 (5)
C4—C5—H5	119.6	C13—C14—H14	126.3
C6—C5—H5	119.6	C15—C14—H14	126.3
C1—C6—C5	119.6 (5)	C16—C15—C14	106.5 (5)
C1—C6—C7	120.3 (4)	C16—C15—H15	126.7
C5—C6—C7	120.1 (5)	C14—C15—H15	126.7
C8—C7—C6	120.2 (5)	C15—C16—O3	110.7 (6)
C8—C7—H7	119.9	C15—C16—H16	124.7
C6—C7—H7	119.9	O3—C16—H16	124.7
C7—C8—C9	120.0 (5)	C11—N1—N2	115.1 (4)
C7—C8—H8	120.0	C12—N2—N1	117.7 (4)
C9—C8—H8	120.0	C9—O1—H1	109.5
O1—C9—C10	122.6 (4)	C12—O2—H22	109.5
O1—C9—C8	115.0 (5)	C16—O3—C13	106.0 (4)
C10—C9—C8	122.4 (5)		
			
C6—C1—C2—C3	−0.1 (7)	C6—C1—C10—C9	2.6 (6)
C10—C1—C2—C3	176.6 (4)	C2—C1—C10—C11	6.2 (6)
C1—C2—C3—C4	−0.2 (8)	C6—C1—C10—C11	−177.3 (4)
C2—C3—C4—C5	0.9 (9)	C9—C10—C11—N1	9.5 (6)
C3—C4—C5—C6	−1.3 (8)	C1—C10—C11—N1	−170.6 (4)
C2—C1—C6—C5	−0.3 (6)	O2—C12—C13—C14	−0.1 (8)
C10—C1—C6—C5	−177.1 (4)	N2—C12—C13—C14	−178.3 (5)
C2—C1—C6—C7	178.0 (4)	O2—C12—C13—O3	175.5 (4)
C10—C1—C6—C7	1.2 (6)	N2—C12—C13—O3	−2.6 (6)
C4—C5—C6—C1	1.0 (7)	O3—C13—C14—C15	−0.9 (5)
C4—C5—C6—C7	−177.3 (5)	C12—C13—C14—C15	175.0 (5)
C1—C6—C7—C8	−3.5 (7)	C13—C14—C15—C16	0.7 (6)
C5—C6—C7—C8	174.8 (5)	C14—C15—C16—O3	−0.1 (7)
C6—C7—C8—C9	1.8 (7)	C10—C11—N1—N2	−177.8 (4)
C7—C8—C9—O1	−177.9 (4)	O2—C12—N2—N1	−1.6 (6)
C7—C8—C9—C10	2.2 (7)	C13—C12—N2—N1	176.5 (4)
O1—C9—C10—C11	−4.3 (6)	C11—N1—N2—C12	−168.4 (4)
C8—C9—C10—C11	175.5 (4)	C15—C16—O3—C13	−0.5 (6)
O1—C9—C10—C1	175.7 (4)	C14—C13—O3—C16	0.9 (5)
C8—C9—C10—C1	−4.5 (6)	C12—C13—O3—C16	−175.7 (4)
C2—C1—C10—C9	−173.9 (4)		

### Hydrogen-bond geometry (Å, °)

**Table:**

*D*—H···*A*	*D*—H	H···*A*	*D*···*A*	*D*—H···*A*
O1—H1···N1	0.82	1.84	2.565 (5)	146
